# Evaluating the Efficiency of the Cobas 6800 System for BK Virus Detection in Plasma and Urine Samples

**DOI:** 10.3390/diagnostics13172860

**Published:** 2023-09-04

**Authors:** Junhyup Song, Sinyoung Kim, Eunmin Kwak, Younhee Park

**Affiliations:** Department of Laboratory Medicine, Severance Hospital, Yonsei University College of Medicine, Seoul 03722, Republic of Korea

**Keywords:** analytical performance, automated assay system, BK virus, Cobas 6800, method comparison

## Abstract

We evaluated the overall performance of the Cobas 6800 BKV test in detecting BK virus (BKV). We examined the imprecision of the Cobas 6800 BKV test and compared the qualitative and quantitative results obtained from the Cobas 6800 BKV test and the Real-Q BKV quantification assay. We assessed 88 plasma and 26 urine samples collected between September and November 2022 from patients with BKV infection using the Real-Q BKV quantitative assay. The lognormal coefficient of variation indicated that the inter-assay precision of the Cobas 6800 BKV test ranged from 13.86 to 33.83%. A strong correlation was observed between the quantitative results obtained using the Cobas 6800 BKV test and the Real-Q BKV quantification assay for plasma samples. The Spearman’s rank correlation coefficients (ρ) for plasma, polymerase chain reaction (PCR) media-stabilized urine, and raw urine samples were 0.939, 0.874, and 0.888, respectively. Our analyses suggest that the Cobas 6800 BKV test is suitable for clinical applications owing to the strong correlation between the results obtained using this test and the Real-Q BKV quantification assay in plasma and urine samples. Furthermore, utilizing fresh raw urine samples can be a viable approach for the Cobas 6800 BKV test as it is less labor- and time-intensive.

## 1. Introduction

The BK virus (BKV) was discovered in 1971 in the urine of a patient who underwent renal transplantation [1]. BKV usually remains latent in the epithelium of the renal tubule or urethra after primary infection in early childhood [2]. The prevalence of latent BKV infection is reportedly 65–90% by the age of ten [3]. Under immunocompromising conditions, BKV multiplies extensively and causes clinical manifestations ranging from tubulointerstitial nephritis to hemorrhagic cystitis [4]. BKV-associated nephritis (BKVAN) is the most frequent clinical reactivation in renal transplant recipients and frequently results in graft rejection [5]. Recent studies have extensively explored the characteristics of BKVAN, along with diagnostic and therapeutic strategies [6,7,8,9,10,11,12,13]. BKVAN occurs in 1.3–4% of all patients who undergo kidney transplantation [14,15]. Furthermore, its occurrence leads to graft loss in up to 50% of those affected [6]. It has been reported that male and old recipients, lymphopenia after kidney transplantation, and a maintenance therapy regimen involving corticosteroids can act as independent risk factors for BKVAN [7,8,16]. Intensive monitoring of BKV reactivation and the preemptive reduction of immunosuppression can improve graft survival in patients with BKVAN [12,17].

Molecular diagnostic methods are widely used to detect and monitor BKV infection. BKV-specific immunohistochemical staining after kidney transplant biopsy is the most definitive method of diagnosing BKVAN [18]. However, molecular assays performed on urine or plasma samples are easier and safer than biopsies and help to avoid false-negative results resulting from biopsy sampling errors [19]. Although most cases of BKV in urine do not progress to viremia or nephritis, BKV replication is typically observed in a sequence from viruria to viremia to nephritis [20,21]. Previous studies have shown that the highest prevalence of BK viruria occurs at two to three months, with viremia typically appearing at three to six months. Therefore, BKV assays on urine samples allow the faster detection of BKV replication than those performed on plasma samples.

Polymerase chain reaction (PCR) is the most widely used and popular molecular assay for the detection of BKV. The diagnosis of BKV infection through PCR was first established in the 1980s [22]. Subsequently, PCR assays have successfully been utilized for the detection of BKV DNA in urine and plasma in renal allograft recipients [23,24]. Furthermore, the quantification of the BKV viral load is achieved through real-time quantitative PCR (qPCR) assays [25]. The quantification of the viral load has enabled the sensitive identification of BKV viremia and the monitoring of the response to therapeutic intervention [21]. Time-serial monitoring of viral replication plays a crucial role in BKV management strategies, involving the reduction of immunosuppression and response monitoring as fundamental measures in managing the risk of BKVAN in transplant recipients [26].

In recent years, the increased demand for molecular diagnostic tests for infectious agents has challenged clinical laboratories, resulting in a need to develop efficient and accurate high-throughput testing platforms. The Cobas 6800 system (Roche Diagnostics) is a fully automated molecular testing instrument [27] based on automated DNA extraction and real-time qPCR, facilitating high-throughput and cost-effective viral testing by allowing the shared use of reagents for various viruses, including cytomegalovirus, hepatitis B virus, hepatitis C virus, Epstein–Barr virus (EBV), human immunodeficiency virus-1/2, human papillomavirus, and influenza A/B. The Cobas BKV test on Cobas 6800 received FDA clearance in September 2020. Although several studies have evaluated its efficiency in detecting various pathogens, data regarding its BKV detection efficacy are limited.

Here, we assessed the performance of the Cobas 6800 BKV test on plasma and urine specimens and compared its results with those obtained from the Real-Q BKV quantification assay (BioSewoom Inc.). Our analyses indicated that the Cobas 6800 BKV test is well suited for clinical applications due to the strong correlation observed between its results and those of the Real-Q BKV quantification assay in both plasma and urine samples. Additionally, employing fresh raw urine samples for the Cobas 6800 BKV test can be a viable approach, as it reduces labor and expedites the turnaround time.

## 2. Materials and Methods

### 2.1. Study Design

This study was conducted between September 2022 and November 2022. We evaluated the imprecision of the Cobas 6800 BKV test based on the Clinical and Laboratory Standards Institute EP15-A3 [28]. Moreover, we assessed the quantitative agreement between the Cobas 6800 BKV test and Real-Q BKV quantification assay using plasma and urine samples. We collected 88 plasma and 26 urine samples from patients with BKV infection confirmed via the Real-Q BKV quantitative assay. These samples were then analyzed using the Cobas 6800 BKV test to compare the efficacy of the two methods. In the urine samples, we measured the BKV loads in two different preparations: (1) raw urine samples and (2) urine samples stabilized in Cobas PCR medium. These measurements were then compared with the results obtained from the Real-Q BKV quantitative assay. This study was approved by the Institutional Review Board of the Severance Hospital, Seoul (IRB Number: 2022-3736-001).

### 2.2. Cobas 6800 BKV Test

The Cobas 6800 system (Roche Diagnostics, Mannheim, Germany) comprises an automated sample preparation module and a PCR amplification and detection module. Automated nucleic acid extraction was performed using a lysis reagent, followed by magnetic bead purification. BKV DNA was selectively amplified using dual BKV-specific primers that target coding regions for the viral protein 2 (VP2) and small t-antigens (Table 1). The manufacturer-provided DNA-quantitation standard (DNA-QS) molecule served as the internal standard for the assay. Two detection probes for BKV sequences and one for DNA-QS labeled with fluorescent reporter dyes were utilized for the real-time detection and analysis of PCR products. The limit of detection (LOD) of the assay was determined via probit analysis of signal detection from a dilution series using the WHO BKV international standard (National Institute for Biological Standards and Control 14/212), ranging from 40.0 to 1.25 BKV DNA IU/mL. Qualitative results were considered positive if BKV DNA loads were higher than the LOD of the assay (21.5 IU/mL and 12.2 IU/mL for plasma and urine samples, respectively).

### 2.3. Cobas PCR Medium

Cobas PCR medium (Roche Diagnostics, Mannheim, Germany) serves as a nucleic-acid-stabilizing transport and storage medium for urine specimens. Based on the manufacturer’s suggestion, once stabilized in the media, the maximum sample storage duration was increased from 24 h to 90 days at 2–30 °C. Specimens were collected according to the manufacturer’s instructions. Briefly, 10–50 mL of first-catch urine was collected in a urine collection cup. Subsequently, 4.5 mL of the urine sample was immediately transferred to a PCR media tube and inverted five times to mix the specimen and media.

### 2.4. Cobas 6800 BKV Test Imprecision

The inter-assay precision was estimated based on CLSI EP15-A3 [28]. The following control materials from two separate origins containing high or low concentrations of the measurand were used for the evaluation: (i) Cobas EBV/BKV control kit (Roche catalog number: #08688214190; Roche Diagnostics, Mannheim, Germany); (ii) AcroMetrix BKV controls (Thermo Fisher Scientific catalog number: #960050–960051; Thermo Fisher Scientific, Waltham, MA, USA). The control materials were diluted in filtered ethylenediaminetetraacetate plasma for measurement.

### 2.5. Real-Q BKV Quantitative Assay

The Real-Q BKV quantification (BioSewoom Inc., Seoul, Korea) assay is a real-time qPCR assay using BKV-specific primers and dual-target TaqMan probes (Thermo Fisher Scientific, Waltham, MA, USA). BKV DNA was selectively amplified using BKV-specific primers, which targeted a 262-bp sequence in the coding region for viral protein 1 (VP1) of BKV (Table 1). The manufacturer’s internal standard was amplified and examined using a TaqMan probe in parallel to discriminate false-negative cases. The amplified product from VP1 was detected using a TaqMan probe labeled with the reporter and quencher dyes FAM and TAMRA, respectively, whereas the amplified product from the internal control was detected using another TaqMan probe labeled with the reporter and quencher dyes VIC and TAMRA, respectively. Qualitative results were reported to be positive for BKV DNA loads higher than 4500 copies/mL, which is equivalent to 1755 IU/mL.

### 2.6. Statistical Analysis

The qualitative agreement between the Cobas 6800 BKV test and Real-Q BKV quantitative assay was analyzed using Cohen’s κ, where κ values > 0.60 indicate substantial to perfect agreement, values between 0.20 and 0.60 suggest fair to moderate agreement, and values < 0.20 represent poor to slight agreement [29]. Specimens with positive results obtained from both assays were quantitatively compared. Relations between the results were analyzed using Passing–Bablok regression. The quantitative agreement between the two assays was analyzed using Spearman’s correlation coefficient (ρ) and illustrated using Bland–Altman plots. All statistical analyses were performed using Analyse-it for Microsoft Excel 5.40 (Analyse-it Software Ltd., Leeds, UK) and SPSS version 26.0 (IBM Corp., Armonk, NY, USA). A *p*-value < 0.05 was considered statistically significant.

## 3. Results

### 3.1. Cobas 6800 BKV Test Imprecision

The inter-assay precision, expressed as the lognormal percent coefficient of variation, ranged from 13.86 to 23.82 for the Cobas 6800/8800 EBV/BKV control kit and from 19.88 to 33.83 for the AcroMetrix BKV controls (Table 2).

### 3.2. Agreement between Qualitative Data from Cobas 6800 BKV Test and Real-Q BKV Quantification Assay of Plasma and Urine Samples

The concordance rate between the two assays was 89.8% for plasma samples, 100.0% for urine samples stabilized in Cobas PCR media, and 100.0% for fresh raw urine samples (Table 3). Similarly, Cohen’s κ values were 0.793 for plasma samples and 1.000 for urine samples stabilized in Cobas PCR media and fresh raw urine samples.

### 3.3. Comparison of Quantitative Data from the Two Assays on Plasma Samples

**Figure 1:**
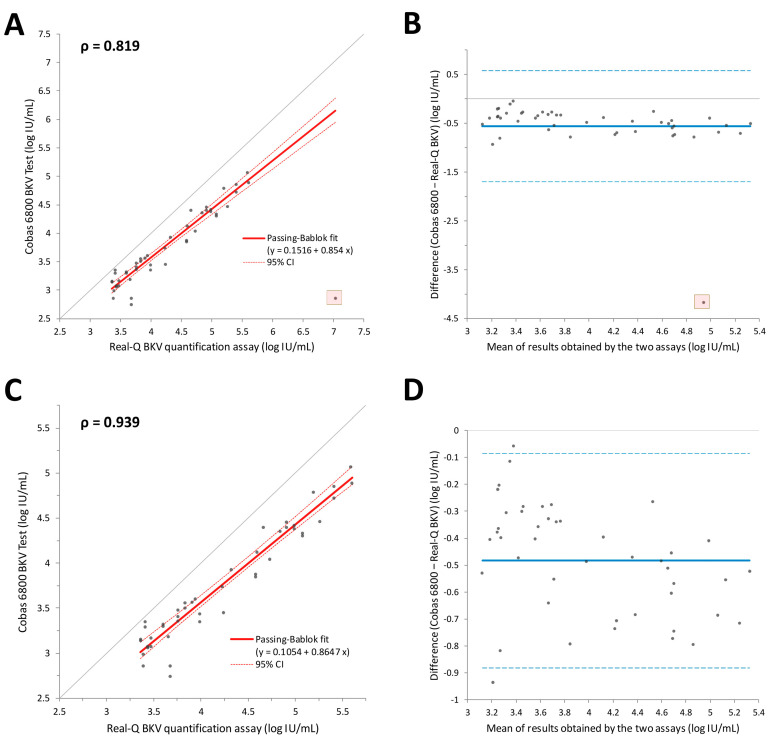
The correlation between the results obtained using the Real-Q BKV quantification assay and Cobas 6800 BKV test using plasma samples is presented as the (**A**) Passing–Bablok regression line and (**B**) Bland–Altman plot. The analyses were performed again after excluding one discrepant result, which is marked with a semitransparent red box in the graphs. The resulting correlation is also represented by a (**C**) Passing–Bablok regression line and (**D**) Bland–Altman plot.

Forty-six plasma samples that tested positive for BKV in both assays were quantitatively compared. Initially, we analyzed all data points, including one discordant result, using Passing–Bablok regression. Spearman’s ρ was relatively low at approximately 0.819, influenced by the discordant result (Figure 1). We then repeated the comparison after excluding the discordant result, to better understand the general tendency without the influence of outliers. We observed a significant correlation between the quantitative results of the two assays (Spearman’s ρ = 0.939). Bland–Altman analysis indicated a mean difference of 0.48 log IU/mL between the two assays.

### 3.4. Comparison of Quantitative Data from the Two Assays on Urine Samples

**Figure 2:**
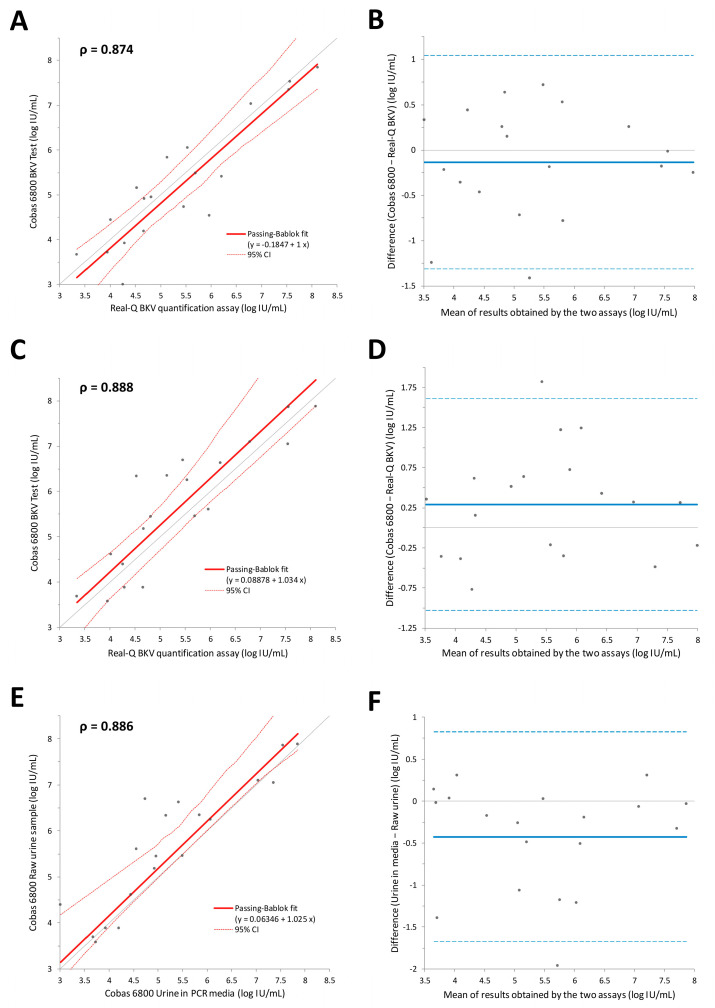
The correlations between the results obtained using the Real-Q BKV quantification assay and the Cobas 6800 BKV test on urine samples prepared using two different methods are presented in the form of Passing–Bablok regression lines (**A**,**C**,**E**) and Bland–Altman plots (**B**,**D**,**F**). (**A**,**B**) The correlation between the results of the Real-Q BKV quantification assay and Cobas 6800 BKV test using urine samples stabilized in Cobas PCR media. (**C**,**D**) The correlation between the results of the Real-Q BKV quantification assay and Cobas 6800 BKV test using fresh raw urine samples. (**E**,**F**) The correlation between the results of the Cobas 6800 BKV test using the samples prepared using two different methods.

A quantitative comparison of 19 urine samples within the analytical measurement range of both assays was performed. We measured BKV loads using urine samples prepared in two ways: urine samples stabilized in Cobas PCR medium immediately after collection and raw urine samples. The Passing–Bablok regression slopes ranged from 1.000 to 1.025, with Spearman’s ρ ranging from 0.874 to 0.888 (Figure 2). Mixing and stabilizing urine samples in PCR media before measurement did not significantly affect the correlation between the assays. Moreover, the results obtained from the urine samples stabilized in PCR media and those from raw urine samples demonstrated an acceptable correlation. The Bland–Altman analysis revealed that raw urine samples generally exhibited higher viral loads, with a mean difference of 0.48 log IU/mL.

## 4. Discussion

BKV infection leads to common complications after renal transplantation owing to viral reactivation [30], requiring frequent monitoring and the timely reporting of viral reactivation for the effective management of kidney transplant recipients. In this study, we evaluated the analytical performance of the Cobas 6800 BKV test. Our findings suggest that the imprecision of the test was within an acceptable range, with lognormal percentage coefficients of variation ranging from 13.86 to 33.83 (Table 2). To avoid an exceedingly optimistic interpretation of the analytical performance, we presented lognormal percent coefficients of variation instead of simple percent coefficients of variation [31]. Our data on the Cobas 6800 BKV test’s imprecision agree with the manufacturer’s specifications and are also compatible with those reported using other BKV detection kits [32,33,34]. Categorical analysis of 88 plasma and 26 urine samples revealed almost perfect agreement between the two assays (Table 3). Although the agreement rate for plasma samples was 89.8%, the discrepancy in the results for some samples could be attributed to the lower limit of the clinically reportable range (4500 copies/mL) set for the Real-Q BKV quantification assay. Of the nine samples that tested negative using the Real-Q BKV quantification assay but positive using the Cobas 6800 BKV test, eight had viral loads below 1755 IU/mL (equivalent to 4500 copies/mL for the Real-Q BKV quantification assay), based on the Cobas 6800 BKV test. The results were perfectly concordant for urine samples, regardless of whether they were stabilized in the PCR media.

A single plasma sample exhibited discordant quantitative results among the 46 plasma samples that tested positive using both assays (Figure 1). Brief clinical information on the patient who provided this sample is presented in Supplementary Table S1. A 35-year-old man underwent renal transplantation on 22 July 2018. Periodic BKV detection assays were conducted as part of the post-transplant monitoring. The initial immunosuppression regimen included 1000 mg CellCept (mycophenolate mofetil) and 10 mg Prograf (tacrolimus hydrate) daily. After the BKV load in the plasma was reported to be 27,500,000 copies/mL on 16 August 2022, the CellCept dosage was reduced to 500 mg per day.

To the best of our knowledge, no previous studies have reported the cross-reactivity of BKV with other pathogens [35]. We hypothesize that the discordance between the quantitative results could be attributed to the difference in the target DNA region between the assays. The BKV DNA includes coding regions for large T- and small t-antigens and the capsid proteins VP1, VP2, and VP3 [21]. Popular target regions for BKV real-time qPCR are large T, small t, VP1, and VP2 coding regions [36,37,38]. The sequence data accumulated for VP1 and large T regions provide a basis for reliable assay results across various BKV genotypes (Table 1) [39,40]. Meanwhile, the VP2 and small t regions are considered attractive targets for BKV assays because of their high level of conservation among isolates [41]. As only one or two mismatches near the 3′-end of the primer can significantly lower the overall sensitivity of the assay, a strain with polymorphisms in both the VP2 and small T target regions could cause a discrepancy [42].

It is important to note that the subtype of BKV can influence the assay results. A recent study by Rogers et al. discovered a correlation between BKV subtypes and viral loads in Western Australia [43]. Specifically, subtype I exhibited a higher incidence of high plasma viral loads, while non-subtype I displayed a higher incidence of low plasma viral loads. The researchers hypothesized that this discrepancy could be attributed to the reduced amplification efficiency of non-subtype I genotypes when using the real-time quantitative PCR kit. Additionally, a recent report by Ratnayake et al. revealed the higher prevalence of BKV subtype II compared to what was previously expected, as the worldwide prevalence of subtype II or III was reported to be rare [44]. This finding emphasizes the importance of ensuring the accuracy of molecular diagnostic assays for BKV across different subtypes.

The clinical significance of accurate molecular diagnostic assays across diverse genomic variations in BKV was further underscored in a recent study by Kien et al. [45]. They identified a specific SNP involving an A to G transition at position 1745 of the VP1 gene, which was observed in 95% of subtype IV strains isolated from the Vietnamese population. This finding, absent in other countries, suggests a unique evolutionary pattern and a mutation specific to a particular geographic region. Furthermore, Gras et al. revealed that BKV subtypes derived from baseline donor kidney biopsies often differ from those obtained from biopsy samples during BKVAN diagnosis [46]. This observation of a genotype switch suggests genetic heterogeneity within clinically prevalent BKV strains, underscoring the necessity of the accurate monitoring of the viral load across varying subtypes.

Regarding urine samples, both raw urine samples and urine samples stabilized in Cobas PCR media, the Cobas 6800 BKV test results showed similar levels of correlation with the Real-Q BKV quantification assay results (Figure 2). Cobas PCR medium serves as a nucleic-acid-stabilizing transport and storage medium for urine specimens based on the protein denaturation effect of guanidine hydrochloride. Furthermore, the media may remove PCR inhibitors from the sample matrix [47]. Our findings demonstrate that reliable results can be obtained when fresh raw urine samples are assessed using the Cobas 6800 BKV test.

The implementation of a fully automated molecular testing instrument in clinical laboratories is likely to significantly improve the processing capacity. Currently, there is no established optimal screening strategy, and approaches vary among individual facilities. However, the following procedures would generally be acceptable: (i) monthly testing for the first three to six months, followed by testing every three months until the end of the first year; (ii) monthly for the first six months, followed by testing every three months for the first two years, then annually until five years [48,49]. A study conducted by Boan et al. demonstrated that fortnightly testing detected BK virus (BKV) in urine substantially earlier (median detection time of 63 days) than testing every three months (median detection time of 97 days) [50]. Additionally, the first positive urine sample had a lower viral load with fortnightly testing (median 3.27 log10 copies/mL) compared to that with three-monthly testing (median 6.71 log10 copies/mL). Therefore, the increased processing capacity of the automated instrument is expected to effectively facilitate the early diagnosis of BK viruria. Although the cost-effectiveness of urine BKV qPCR has been questioned due to the low rate of progression from viruria to viremia or the eventual development of BKVAN, it could still be utilized as a more sensitive screening test before conducting plasma qPCR, which often requires additional phlebotomy [51].

This study had several limitations. First, comparisons between assays using plasma and urine samples were conducted separately. The simultaneous collection of both sample types could allow the assessment of the concordance between the results from the different sample types. Second, a third PCR assay for plasma samples with discrepant results was not performed. An additional PCR assay using primers targeting other BKV DNA regions, like the small t region, could have resolved the observed discrepancy.

In conclusion, we validated the precision of the Cobas 6800 BKV test and compared its results to the Real-Q BKV quantification assay results. For both plasma and urine samples, the results of the two assays were strongly correlated. For fresh raw urine samples and urine samples stabilized in Cobas PCR medium, the Cobas 6800 BKV test results exhibited similar levels of correlation with the Real-Q BKV quantification assay results. Hence, we demonstrated that the Cobas 6800 BKV test is suitable for clinical use.

## Figures and Tables

**Table 1:** Characteristics of the Cobas 6800 BKV test and Real-Q BKV quantification assay. The contents of this table are based on the manufacturers’ product specifications.

	Cobas 6800 BKV Test	Real-Q BKV Quantification Assay *
Principle	Real-time quantitative PCR	Real-time quantitative PCR
Sample type	Plasma and urine	Serum, plasma, and urine
Sample volume		
Plasma	375 uL	320 uL
Urine	575 uL	320 uL
Total duration	~180 min	~280 min (120 min for DNA extraction, 40 min for pipetting, and 120 min for amplification)
Hands-on time	-	~40 min (thawing reagents and aliquoting and mixing)
Assay targets	VP2 and small t-antigen	VP1
Subtypes detected	Ia, Ic, II, III, and IV	Not specified
Limit of detection		
Plasma	21.5 IU/mL	71.4 IU/mL (183 copies/mL)
Urine	12.2 IU/mL	Not specified
Linear range		
Plasma	21.5 IU/mL to 1 × 10^8^ IU/mL	195 IU/mL to 1.95 × 10^12^ IU/mL (500 copies/mL to 5 × 10^12^ copies/mL)
Urine	200 IU/mL to 1 × 10^8^ IU/mL	Not specified
Imprecision		
Plasma	6.92–25.74%CV	11.58%CV
Urine	4.61–11.55%CV	Not specified

* Analytical performance of the Real-Q BKV quantification assay was converted from copies/mL to IU/mL, using the conversion factor (0.39) provided by the manufacturer. Abbreviations: BKV, BK virus; CV, coefficient of variation; PCR, polymerase chain reaction; VP, viral protein.

**Table 2:** Within-laboratory precision results of the Cobas 6800 BKV test.

Standard Materials’	Mean (Log IU/mL)	SD (Log IU/mL)	Lognormal %CV *
Measurand Levels			
Cobas 6800/8800 EBV/BKV control kit			
High	6.10	0.06	13.86
Low	2.86	0.10	23.82
AcroMetrix BKV control materials			
High	4.95	0.14	33.83
Low	3.52	0.09	19.88

* Lognormal percent coefficient of variation was calculated as sqrt (10^[SD^2 × ln(10)]^ − 1) × 100. Abbreviations: BKV, BK virus; CV, coefficient of variation; EBV, EB virus; SD, standard deviation.

**Table 3:** Qualitative agreement between Cobas 6800 BKV test and Real-Q BKV quantification assay results.

	Real-Q BKV Quantification Assay					
Cobas 6800 BKV Test	Plasma Samples		Urine Samples in PCR Media		Fresh Raw Urine Samples	
	Negative	Positive	Negative	Positive	Negative	Positive
Negative	33	0	4	0	4	0
Positive	9	46	0	22	0	22
Concordance rate, %	89.8		100.0		100.0	
κ	0.793		1.000		1.000	

Abbreviations: BKV, BK virus; κ, Cohen’s kappa; PCR, polymerase chain reaction.

## Data Availability

The data are available from the authors upon reasonable request. Please contact younheep@yuhs.ac with any questions or requests.

## Supplementary Materials

The following supporting information can be downloaded at: https://www.mdpi.com/article/10.3390/diagnostics13172860/s1, Table S1: Clinical characteristics of the patient with discordant plasma viral loads.
