# Supplementary material for: Evaluating the Efficiency of the Cobas 6800 System for BK Virus Detection in Plasma and Urine Samples
Source: Diagnostics (Basel). 2023 Sep 4;13(17):2860. doi: 10.3390/diagnostics13172860 (PMC10487002; doi:10.3390/diagnostics13172860)
Supplement: Supplementary file 1 [file diagnostics-13-02860-s001.zip › diagnostics-2466490-supplementary.pdf]

Supplementary Table S1. Clinical characteristics of the patient with discordant plasma viral loads

PID	Plasma viral loads (IU/mL)		Sex	Age	Diagnosis	Basis for BKV test	Trends in BK viral loads in plasma
	Real-Q	Cobas 6800					
P-45	10,725,000 (27,500,000 copies/mL)	707	M	35	Z94.0: Kidney transplant status	The patient received a kidney transplant on July 22, 2018, owing to end-stage renal disease that progressed from focal segmental glomerulosclerosis	Dec 13, 2022: 14,300 copies/mL<br>Nov 15, 2022: 7,220 copies/mL<br>Aug 16, 2022*: 27,500,000 copies/mL<br>Jul 29, 2021: <4,500 copies/mL<br>Mar 26, 2021: <4,500 copies/mL

*The plasma sample for comparison between the assays was collected on August 16, 2022.

Abbreviations: BKV, BK virus; IU, international unit; PID, patient identification.
